# A closer look at students’ knowledge of effective learning strategies, where they learn about them, and why they do not use them

**DOI:** 10.1186/s41235-025-00693-8

**Published:** 2025-12-08

**Authors:** Shana K. Carpenter, Christopher A. Sanchez

**Affiliations:** School of Psychological Science, Oregon State University, Reed Lodge, 2950 SW Jefferson Way, Corvallis, OR 97331 USA

**Keywords:** Study strategies, Learning, Metacognition, Self-regulated learning, Motivation

## Abstract

The current survey measured students’ knowledge, use, and perceived effectiveness of different learning strategies. Whereas a common assumption from previous research is that students forego using effective strategies because they lack awareness of them, the current results demonstrate that students are quite aware of certain strategies such as retrieval practice, spacing, and explaining, but are less aware of other strategies like pretesting and interleaving, and seem to overrate the effectiveness of highlighting and rereading notes. Even when students were aware of effective learning strategies, their primary reasons for not using them were the cognitive costs associated with them (time, effort, anxiety, and increased planning and preparation). Students reported teachers, friends, and self-discovery as the most common sources of learning about both effective and ineffective strategies, and published research as the least common source. We discuss the implications of these results for designing interventions aimed at improving students’ self-regulated learning decisions.

**Supplementary Information:**

The online version contains supplementary material available at 10.1186/s41235-025-00693-8.

## Introduction

Successful learning depends on good study behaviors. The increasing flexibility of modern education brings greater autonomy for students, and as a result, greater need for students to manage their own learning. Doing so requires making the right decisions about how and when to engage with material in ways that promote learning.

Unfortunately, the study decisions students make often misalign with empirical evidence on the best ways to learn. For example, despite widespread benefits of spacing, students prefer to “cram” their studying into shorter time periods right before exams (e.g., Geller et al., 2018; Hartwig & Dunlosky, 2012). Given the real-world consequences of such poor study decisions, it is important to identify the underlying reasons for these decisions. Though previous research has reported some study strategies that students use, it has not thoroughly reported the reasons why students do *not* use the most effective strategies, nor has it explored exactly where students get their knowledge about these strategies.

Accordingly, the current study explored students’ knowledge of several study strategies, where they learn about them, and why they forego using them. Below we summarize the research to date on the effectiveness and self-reported use of each strategy (see Table 1).

**Table 1:** Learning strategies explored in the current study

Strategy	Brief description
Retrieval practice	Retrieving information from memory in the form of practice questions, flashcards, or free recall
Pretesting	Answering questions about to-be-learned material prior to learning that material
Spacing	Separating out study sessions across different points in time
Interleaving	Mixing together study of different but related concepts within the same learning session
Examples	Generating examples of how material applies to one’s own life
Explanation	Explaining the material to oneself or someone else
Summarizing	Creating summaries of material being learned
Rereading	Reading textbook or notes over again after initial reading
Recopying notes	Creating a typed or written copy of one’s own notes
Highlighting	Highlighting or underlining information in a text while reading
Writing notes	Writing or typing notes based on information in a lecture or text

### Learning strategies and how often students use them

#### Retrieval practice

Despite the well-known benefits of retrieval practice (for reviews, see Adesope et al., 2017; Carpenter et al., 2022; Pan & Rickard, 2018; Rowland, 2014; Yang et al., 2021), students report using retrieval primarily as a way to check their knowledge rather than a direct learning strategy (Hartwig & Dunlosky, 2012; Kornell & Bjork, 2007; Morehead et al., 2016; Yan et al., 2014). Students also forego using retrieval when given the option, preferring to reread material than to retrieve it (Kirk-Johnson et al., 2019; Tullis & Maddox, 2020).

#### Pretesting

Answering questions prior to learning significantly enhances learning (for a recent review, see Pan & Carpenter, 2023), and is more beneficial than reading learning objectives (Sana et al., 2020). However, pretesting as a self-reported learning strategy has not been explored as often as some of the other strategies in Table 1. Thus, the current study provides new data on students’ use of pretesting.

#### Spacing

Spacing study across days or weeks benefits learning (for reviews, see Carpenter & Pan, in press; Cepeda et al., 2006), but students nonetheless prefer to cram their studying (Geller et al., 2018; Hartwig & Dunlosky, 2012). Dirkx et al. (2019) found that students reported using spacing less than 5% of the time (see also Zung et al., 2022). Observational studies have also shown that the most common time for students to access online study tools is immediately before exams (Corral et al., 2020; Lui et al., 2019).

#### Interleaving

Studying a mixture of different but related concepts is beneficial, particularly for learning to differentiate concepts that might otherwise be confused (e.g., Kornell & Bjork, 2008; Rohrer et al., 2020; Samani & Pan, 2021). When asked which schedule they prefer for different but related concepts, however, students are more likely to block by concept rather than interleave (Abel et al., 2024; Carvalho et al., 2016; Tauber et al., 2013).

#### Rereading and recopying notes

Students often reread course materials and recopy notes (Geller et al., 2018; Hartwig & Dunlosky, 2012; McAndrew et al., 2015; Yan et al., 2014). Although popular, these are relatively passive strategies that do little to help learning. Though rereading produces some small gains (Callender & McDaniel, 2009), the gains produced by retrieval practice far outweigh those of rereading (e.g., Dunlosky et al., 2013) or recopying (Carpenter & DeLosh, 2005).

#### Highlighting or underlining

Highlighting or underlining text are also popular (Geller et al., 2018; Hartwig & Dunlosky, 2012; McAndrew et al., 2015; Yan et al., 2014), but produce minimal learning gains (Ponce et al., 2022). Studies show a wide range in students’ abilities to select relevant information to highlight, and in the absence of organizational awareness or training that can help with this, highlighting is generally no more effective than simply reading (Dunlosky et al., 2013; Mason et al., 2024).

#### Note-taking

Over 95% of students report taking notes (Morehead et al., 2019; Palmatier & Bennett, 1974), and students commonly rely on notes to study (Morehead et al., 2016; Nandagopal & Ericsson, 2012; Van Meter et al., 1994). Though taking notes over a lecture aids learning more than merely listening (Kobayashi, 2005), the effectiveness of note-taking depends on students’ selection of relevant information (Kiewra et al., 1989; Northern et al., 2023; Titsworth, 2004).

#### Explaining, summarizing, and generating examples

The remaining strategies fall under the umbrella of generative or constructive learning activities.^1^ Explaining the material to oneself or someone else benefits learning compared to extra study time (Fiorella et al., 2020; Hoogerheide et al., 2016), or merely preparing to explain (Fiorella & Mayer, 2014; Hoogerheide et al., 2014). Writing summaries benefits learning more than copying (Bretzing & Kulhavy, 1979; Oded & Walters, 2001), and generating examples benefits learning more than extra study time (Obergassel et al., 2025; Rawson & Dunlosky, 2016). These strategies are more effective when explanations include elaborations and links to prior knowledge (Roscoe, 2014), when summaries include the main points relevant for understanding (Bednall & Kehoe, 2011; Dunlosky et al., 2013), and when examples accurately illustrate the concepts (Rawson & Dunlosky). The research so far suggests that these strategies are not widely used, as Zepeda and Nokes-Malach (2021) found that less than 15% of students reported using these strategies.

### Why do students not use effective learning strategies?

There are likely multiple reasons why students do not use effective strategies. These reasons are currently not well-understood. It has long been assumed that students lack awareness of effective strategies, based on studies showing that students often endorse massing over spacing (Cohen et al., 2013; Emeny et al., 2021; Wissman et al., 2012), blocking over interleaving (Hartwig et al., 2022; McCabe, 2011; Yan et al., 2016, 2017), and rereading over retrieval (Agarwal et al., 2008; McCabe; Tullis & Maddox, 2020; Yeo & Fazio, 2019).

Other research, however, suggests that effort plays a role. Effective strategies involve more effort than ineffective strategies, and though these “desirable difficulties” are good for learning, they can be offputting for students (Bjork & Bjork, 2023) or interpreted as a sign of failed learning (Janssen et al., 2023; Kirk-Johnson et al., 2019; Onan et al., 2022; Tullis & Maddox, 2020). Although a recent study by Rea et al. (2022) found that time and effort were students’ primary reasons for not using a group of active strategies (including retrieval practice and spacing), students’ reasons for not using each individual strategy were not collected.

### Current study

The current study addresses three unanswered questions. First, why do students not use effective learning strategies? We asked students two questions about the strategies in Table 1: (1) “*How effective do you believe this strategy is for learning*?” and (2) “*What are the main reasons why you might not use this strategy?*” This provides the first known data on students’ reasons for not using a given strategy even when they know it is effective.

Second, do students more often utilize strategies they believe are effective? Students use ineffective strategies, and they often believe that ineffective strategies are good for learning, however these two lines of research have been explored separately. Thus, we included a third question: (3) “*In your own studying, how often do you use the following strategy?*” Combined with Question #1, this allows a direct analysis of whether the frequency with which students use particular strategies correlates with their perceived effectiveness of those strategies.

Third, where do students learn about the strategies? The majority of students report that their study behaviors were not taught to them (Geller et al., 2018; Hartwig & Dunlosky, 2012; Kornell & Bjork, 2007; Yan et al., 2014), which raises questions about where students acquire this knowledge. To provide new data on where students learn about individual study strategies, we included a fourth question: (4) “*Please indicate whether or not you had knowledge of this strategy prior to completing this survey. If so, where did you learn about the strategy?*”.

### Method

#### Participants

The survey was completed online by 201 undergraduate students at Oregon State University (OSU) for introductory psychology course credit. It was accessible to students during the fall academic quarter (10 weeks beginning in October and ending in December, 2024), and the sample represents the number of students who completed the survey during that time. The study was approved by the Institutional Review Board (IRB) of OSU and was not preregistered. Raw data are available here: https://osf.io/kpua4/?view_only=0f09de334c3b450281081ed0890f3ec5.

### Materials, design, and procedure

Students read the informed consent document, then clicked a button to begin the survey. Questions (provided in the Appendix) were shown one at a time, with the strategies listed in a unique random order.^2^ For the first question, students were presented the instructions “*Think about the studying that you do for your classes. In your own studying, how often do you use each of the following strategies?*” and the response options 1–5 (see Appendix). Unlike previous studies using a more general question where students checked off strategies they use (e.g., Hartwig & Dunlosky, 2012), the current question provides more nuanced usage data for each individual strategy.

The second question was similar in purpose to Rea et al.’s (2022) question about why students do not use a group of strategies. However, we designed it so that students reported specific reasons for not using each individual strategy. Students saw the instructions “*Below we have listed each of those strategies again. What are the main reasons why you might NOT use each of these strategies?*” with the response options (a) through (l) (see Appendix).

For the third question, created for the current survey, students saw the instructions “*Below we have listed each of those strategies again. This time, please indicate whether or not you had knowledge of these strategies prior to completing this survey. If so, where did you learn about the strategy?*” with the response options (a) through (j) (see Appendix).

For the fourth question, students were asked about their perceived effectiveness of each strategy. Although previous studies have asked students to compare the effectiveness of two strategies (Kornell & Bjork, 2008), the current question provides more detailed ratings for each individual strategy. Students saw the instructions “*Below we have listed each of those strategies, one last time. Please indicate how effective you believe each strategy is for learning. For each strategy, use the sliding scale to choose a number between 1 (not at all effective) and 10 (highly effective)*.” The sliding scale was set to a default value of 1.

On the final screen, students answered three questions about themselves (their academic major, gender identity, and how they typically perform academically), then read a debriefing statement.

## Results and discussion

Nine students began the survey but did not finish it. Data from four additional students who had completion times (and largely invariable responses) within the three-minute range were excluded,^3^ as were data from three additional students who took more than two hours to complete the survey. All analyses were based on the remaining 185 students (131 women, 49 men, 5 other). Below we present the full sample results. Analyses by student major are in the supplemental material.

### How often do students use the strategies, and how effective do they perceive them to be?

Table 2 lists the percentage of students reporting how often they use each strategy. The most popular strategies were rereading, highlighting, and note-taking. Recopying notes was least popular. Though more than half of students indicated that they use retrieval practice often or almost always, other effective strategies (summarizing, explaining, spacing, interleaving, and generating examples) were used less often. Pretesting was the effective strategy used least often.

**Table 2:** How often students report using each learning strategy

	Never	Rarely	Sometimes	Often	Almost always
Reread	1%	6%	28%	35%	31%
Highlight	7%	14%	19%	32%	28%
Write notes	3%	4%	14%	29%	50%
Summaries	9%	26%	30%	26%	9%
Recopy	30%	29%	24%	9%	8%
Explain	5%	18%	38%	27%	12%
Retrieve	5%	13%	26%	32%	24%
Pretest	24%	25%	22%	18%	11%
Space	4%	18%	37%	25%	16%
Interleave	6%	17%	38%	28%	10%
Examples	3%	11%	39%	29%	18%

These results are consistent with those of previous studies showing that students often use retrieval, rereading, and highlighting, but less often use spacing and recopying notes (Geller et al., 2018; Hartwig & Dunlosky, 2012; Yan et al., 2014). They also provide new data on how often students use note-taking, summaries, explanations, pretesting, interleaving, and examples.

**Fig. 1:**
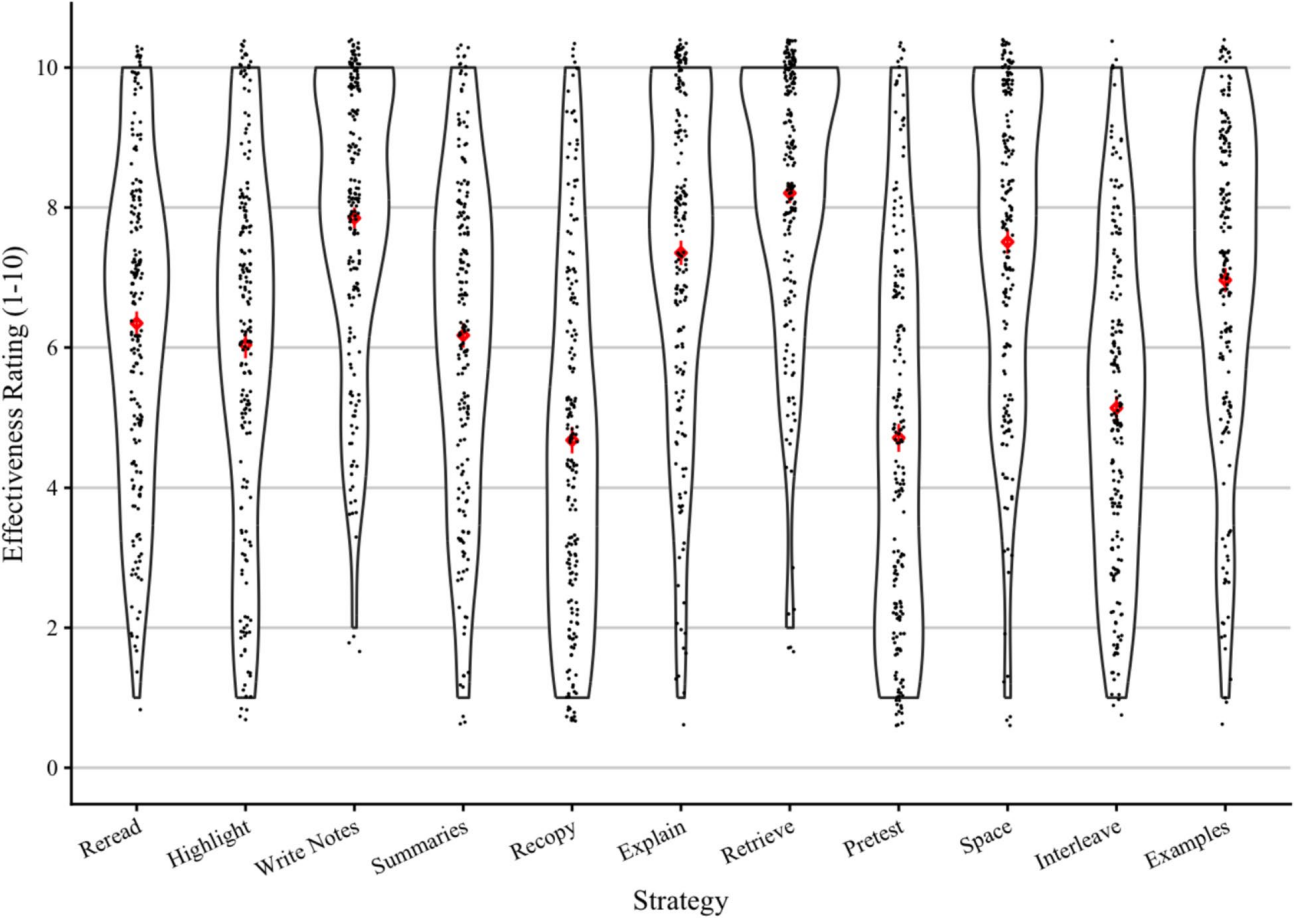
Violin plots of students’ reported effectiveness ratings for each strategy. *Note*: Mean and standard error are shown for each strategy

Figure 1 shows students’ effectiveness ratings for each strategy. Students had some awareness of the benefits of retrieval, spacing, and explaining. However, other effective strategies (summarizing and generating examples) were rated similarly to the ineffective strategies rereading and highlighting. Although students appeared to have some awareness of the ineffectiveness of recopying notes, they gave similar ratings to pretesting and interleaving. These results are consistent with those of Rea et al. (2022), who found that students recognized retrieval, spacing, and explanations (but not interleaving) as strategies likely to be utilized by high-achieving students. They also highlight summarizing, generating examples, pretesting, and interleaving as strategies that may not be recognized as effective.

For each strategy, we correlated each student’s effectiveness rating with how often they use that strategy (verbal responses converted such that “*never*” = 1, “*rarely*” = 2, “*sometimes*” = 3, “*often*” = 4, and “*almost always*” = 5). These correlations were significantly positive (all *p*s < .001) across all strategies:^4^ rereading (*r* = .35), highlighting (*r* = .67), note taking (*r* = .36), summarizing (*r* = .30), recopying notes (*r* = .54), explaining (*r* = .49), retrieval (*r* = .45), pretesting (*r* = .47), spacing (*r* = .31), interleaving (*r* = .46), and generating examples (*r* = .62). Thus, when students have a stronger belief that a strategy works, they use it more often.

### Why do students forego using the strategies?

Table 3 reports students’ reasons for not using each strategy. For effective strategies, students most often listed too much planning or preparation, effort, and feeling anxious, nervous, or stressed. About a third of students indicated that they do not use pretesting and interleaving because those strategies do not help learning. Thus, though students may have some misconceptions about pretesting and interleaving, common reasons for not using effective strategies are tied to their perceived cognitive costs (planning, preparation, anxiety, and effort). These data align with others showing that students’ use of active and effective strategies can be inhibited by time (Biwer et al., 2020), anxiety, and effort (Hui et al., 2022; Rea et al., 2022).

**Table 3:** Percentage of students indicating reasons why they might not use each strategy

	Reread	Highlight	Notes	Summaries	Recopy	Explain	Retrieve	Pretest	Space	Interleave	Examples
Too much time	70%	28%	55%	50%	78%	24%	36%	30%	50%	27%	17%
Does not help my learning	18%	36%	8%	20%	30%	15%	5%	32%	8%	28%	25%
Anxious, nervous, stressed	11%	4%	10%	6%	7%	37%	26%	28%	19%	29%	14%
Too difficult	8%	3%	8%	12%	9%	10%	12%	11%	14%	21%	16%
Not interesting	32%	18%	15%	22%	23%	10%	9%	14%	9%	8%	18%
Don’t know how to use it	3%	6%	2%	11%	4%	4%	8%	12%	5%	10%	17%
Not interested in learning	8%	7%	9%	5%	4%	5%	7%	4%	5%	5%	4%
Do not need it	5%	16%	8%	10%	13%	11%	5%	11%	6%	13%	14%
New strategy	2%	2%	3%	10%	6%	7%	5%	12%	6%	12%	10%
Too much effort	32%	21%	35%	37%	46%	21%	29%	19%	29%	17%	17%
Too much planning or prep	9%	4%	9%	12%	8%	18%	35%	28%	57%	19%	6%
Other	5%	10%	11%	5%	3%	17%	5%	5%	8%	6%	11%

Most of the time, students used the “other” category to indicate that they do use the strategy. Occasionally they used “other” to write in circular responses that were already among the response options (e.g., it takes a lot of time; it is not very effective), and in those cases the responses were counted within those relevant response options instead of in the “other” category. "Other" responses indicating alternative reasons for not using the strategies included not having a highlighter (as a reason for not using highlighting), hands getting tired (as a reason for not taking notes), and not having anyone to explain to (as a reason for not explaining the material to someone else). Students could choose more than one response for each strategy, so percentages do not sum to 100

Students’ reasons for not using ineffective strategies had more to do with something the strategies were lacking. About one third of students forego rereading because they do not find it interesting, and about one third forego highlighting and recopying notes because they do not help learning. Finally, about one third forego rereading and recopying notes because these strategies take too much effort.^5^ The most popular reason for not using many of the strategies is that they take too much time, which may indicate that students perceive time constraints as a general barrier to studying.

### Why do students not use effective learning strategies even when they know the strategies are effective?

We restricted our analysis to students giving effectiveness ratings of 9 or 10 (on the 1–10 scale) for each effective strategy, and examined these students’ reasons for not using each strategy.^6^ The data for the effective strategies are in Table 4, with sample sizes in parentheses.

**Table 4:** Students’ reasons for not using effective strategies that they rated as highly effective

	Summaries(*n* = 29)	Explain (*n* = 45)	Retrieve (*n* = 88)	Pretest (*n* = 18)	Space (*n* = 67)	Interleave (*n* = 13)	Examples (*n* = 47)
Too much time	45%	33%	35%	39%	54%	54%	26%
Does not help my learning	17%	0%	5%	22%	1%	0%	13%
Anxious, nervous, stressed	3%	47%	27%	17%	22%	31%	13%
Too difficult	0%	9%	16%	17%	9%	23%	21%
Not interesting	7%	7%	7%	22%	10%	0%	19%
Don’t know how to use it	7%	2%	7%	17%	3%	8%	15%
Not interested in learning	10%	4%	8%	6%	1%	15%	6%
Do not need it	10%	2%	2%	6%	4%	0%	9%
New strategy	7%	7%	6%	6%	1%	0%	9%
Too much effort	41%	29%	36%	33%	16%	23%	30%
Too much planning or prep	14%	33%	38%	17%	55%	15%	9%

As in the full dataset, time and cognitive costs appear to be the primary barriers. Even when students know these strategies work, they forego using them due to the anxiety, effort, and degree of planning and preparation required.

These data rule out the common assumption that lack of awareness accounts for students not using effective strategies. This could explain why merely informing students about effective strategies does not increase their use (Broeren et al., 2021; Carpenter, 2023; Rea et al., 2022; Simone et al., 2023). Interventions would thus more likely be successful if they can reduce the time and cognitive costs associated with using effective strategies.

### Where do students acquire knowledge about learning strategies?

Table 5 reports where students learned about each strategy. At least one third of students reported learning the strategies from a teacher. Students also listed their own experience and friends as common sources. The least common sources were published research and academic support centers. With the exception of interleaving, students rarely indicated that they had never learned about the strategies.

**Table 5:** Percentages of students indicating where they learned about each strategy

	Reread	Highlight	Notes	Summaries	Recopy	Explain	Retrieve	Pretest	Space	Interleave	Examples
Never learned	1%	2%	1%	10%	17%	8%	1%	16%	3%	28%	12%
Teacher	60%	60%	70%	55%	31%	45%	72%	60%	68%	36%	46%
Tutor	10%	17%	13%	9%	8%	11%	15%	10%	12%	4%	6%
Friends	21%	29%	28%	12%	19%	32%	32%	13%	17%	9%	12%
Social media	7%	9%	12%	7%	9%	12%	14%	5%	11%	7%	8%
Research	4%	4%	8%	3%	4%	9%	10%	6%	16%	8%	9%
Academic center	4%	5%	8%	6%	5%	4%	11%	3%	15%	4%	5%
Own experience	48%	36%	45%	23%	28%	42%	34%	19%	32%	20%	36%
Do not remember	9%	11%	9%	10%	15%	12%	8%	8%	8%	14%	11%
Other	1%	1%	1%	1%	1%	1%	1%	2%	2%	0%	1%

Students listed parents as a common “other” response, and sometimes also listed TV. Occasionally students used “other” to write in circular responses that were already among the response options (e.g., I just tried it on my own), and in those cases the responses were counted within those relevant response options instead of in the “other” category. Students could choose more than one response for each strategy, so percentages do not sum to 100

Though previous research has not explored such a comprehensive list of strategies, teachers have been mentioned as a source of acquiring knowledge about learning. Morehead et al. (2019) found that 47% of university students reported learning note-taking skills from a teacher, and Wissman et al. (2012) found that 76% of university students reported receiving general study advice from a teacher. The current study shows that students acquire knowledge on their own as well. The fact that students listed teachers and their own experience as common sources for learning about both effective and ineffective strategies (e.g., Morehead et al., 2016, found that university instructors had good knowledge of spacing and retrieval practice, but strong misconceptions about interleaving) underscores the importance of ensuring that both teachers and students are equipped with accurate knowledge about these strategies. In the current survey academic support centers were an uncommon source (see McCabe, 2018, for data on strategy recommendations from academic support centers), however, it is possible that students in our sample (most likely to be students in their first term at a university) had not yet experienced academic support centers. An open question for future research is how students’ study strategies develop over time and with more academic experience.

In conclusion, the current survey provides new data that help us better understand students’ learning strategies. Students are aware of some effective strategies (retrieval practice, spacing, and explaining), but less aware of others (pretesting and interleaving). Even when students know a strategy works, the cognitive costs associated with it are the primary barriers to using it. Though teachers are the most common source of learning about strategies, students also reported learning about them on their own. Understanding how this self-discovery happens, increasing awareness of the lesser-known effective strategies, and designing interventions that mitigate the barriers to using effective strategies, are worthwhile areas for future research.

### Significance statement

Using good study strategies is critical to successful learning. Despite over 100 years of research on the strategies that consistently enhance learning, the study decisions that students make often run counter to the empirical evidence, showing a tendency for students to use ineffective study strategies instead of effective ones. To understand the reasons behind these decisions, the current survey presented students with several study strategies (both effective and ineffective ones), and asked students to rate how effective they believed each strategy to be, how often they use it, reasons why they might not use it, and where they learned about the strategy. Students showed fairly good knowledge of the effectiveness of retrieval practice, spacing, and explaining, but were less aware of the effectiveness of pretesting and interleaving. Even when students showed good awareness of the effective strategies, the most common reasons for not using those strategies included time costs, anxiety, effort, and increased planning and preparation. Thus, even when students know that a strategy works, they are still likely to forego using that strategy if it comes with high cognitive costs. Students listed teachers as the most common source from which they learned about the strategies, and published research as the least common source. Teachers and self-discovery were the most common source from which students learned about both effective and ineffective strategies, underscoring the need for both teachers and students to have accurate and complete knowledge of the effectiveness of a variety of learning strategies.

## Supplementary Information

Additional file 1.

## Data Availability

The raw data for this study have been made available and can be accessed here: https://osf.io/kpua4/?view_only=0f09de334c3b450281081ed0890f3ec5

## Appendix 1: Survey questions used in the current study

1. Think about the studying that you do for your classes. In your own studying, how often do you use each of the following strategies? (chose one response for each strategy).

- Re-read over textbooks, notes, or lecture materials.
- Highlight or underline material in texts or notes.
- Write notes from the textbook or lectures.
- Create my own summaries of the material that I am learning.
- Re-copy my notes.
- Explain the concepts that I am learning to somebody else.
- Test myself over the material (by using practice questions, flashcards, etc.) to recall information that I have learned without looking at my notes or textbook.
- Test myself before I have learned the material (by using practice questions, flashcards, etc.) to see how much I know about a topic before learning it.
- Space out my studying across multiple days or weeks prior to the exam.
- Study a mixture of different but related concepts within the same study session, rather than one concept at a time.
- Think of ways that the material applies to examples from my own life.

*Note.* Under each strategy, students were presented with the response options 1 = Never, 2 = Rarely, 3 = Sometimes, 4 = Often, 5 = Almost always. The order in which the strategies were presented was randomized for each student.

**2.** Below we have listed each of those strategies again. What are the main reasons why you might NOT use each of these strategies? (For each strategy, check all that apply).

- Re-read over textbooks, notes, or lecture materials.
- Highlight or underline material in texts or notes.
- Write notes from the textbook or lectures.
- Create my own summaries of the material that I am learning.
- Re-copy my notes.
- Explain the concepts that I am learning to somebody else.
- Test myself over the material (by using practice questions, flashcards, etc.) to recall information that I have learned without looking at my notes or textbook.
- Test myself before I have learned the material (by using practice questions, flashcards, etc.) to see how much I know about a topic before learning it.
- Space out my studying across multiple days or weeks prior to the exam.
- Study a mixture of different but related concepts within the same study session, rather than one concept at a time.
- Think of ways that the material applies to examples from my own life.

*Note.* Under each strategy, students were presented with the response options (a) It takes too much time, (b) It does not help my learning, (c) It makes me feel anxious, nervous, or stressed, (d) It is too difficult to use, (e) It is not interesting to me, (f) I do not know how to use this strategy, (g) I am not interested in learning the material, (h) I do not need to use this strategy, (i) This is a new strategy for me, and I do not wish to incorporate a new strategy into my studying, (j) It takes too much effort, (k) It takes too much planning or preparation, (l) Other (please describe). The order in which the strategies were presented was randomized for each student.

3. Below we have listed each of those strategies again. This time, please indicate whether or not you had knowledge of these strategies prior to completing this survey. If so, where did you learn about the strategy? (For each strategy, check all that apply).

- Re-read over textbooks, notes, or lecture materials.
- Highlight or underline material in texts or notes.
- Write notes from the textbook or lectures.
- Create my own summaries of the material that I am learning.
- Re-copy my notes.
- Explain the concepts that I am learning to somebody else.
- Test myself over the material (by using practice questions, flashcards, etc.) to recall information that I have learned without looking at my notes or textbook.
- Test myself before I have learned the material (by using practice questions, flashcards, etc.) to see how much I know about a topic before learning it.
- Space out my studying across multiple days or weeks prior to the exam.
- Study a mixture of different but related concepts within the same study session, rather than one concept at a time.
- Think of ways that the material applies to examples from my own life.

*Note.* Under each strategy, students were presented with the response options (a) I have never learned about this strategy, (b) I learned about this strategy from a teacher, (c) I learned about this strategy from a tutor, (d) I learned about this strategy from friends, (e) I learned about this strategy from social media, (f) I learned about this strategy from research published in articles or books, (g) I learned about this strategy from an academic support center, (h) I discovered this strategy through my own experience, (i) I know about this strategy, but I do not remember where I learned about it, (j) Other (please describe). The order in which the strategies were presented was randomized for each student.

4. Below we have listed each of those strategies, one last time. Please indicate how effective you believe each strategy is for learning. For each strategy, use the sliding scale to choose a number between 1 (not at all effective) and 10 (highly effective).

- Re-read over textbooks, notes, or lecture materials.
- Highlight or underline material in texts or notes.
- Write notes from the textbook or lectures.
- Create my own summaries of the material that I am learning.
- Re-copy my notes.
- Explain the concepts that I am learning to somebody else.
- Test myself over the material (by using practice questions, flashcards, etc.) to recall information that I have learned without looking at my notes or textbook.
- Test myself before I have learned the material (by using practice questions, flashcards, etc.) to see how much I know about a topic before learning it.
- Space out my studying across multiple days or weeks prior to the exam.
- Study a mixture of different but related concepts within the same study session, rather than one concept at a time.
- Think of ways that the material applies to examples from my own life.

*Note.* Under each strategy, students were presented with a sliding scale that they could move from 1 to 10. The order in which the strategies were presented was randomized for each student.

5. Please indicate your current academic major(s):

6. Please indicate your gender identity: (a) female, (b) male, (c) other.

7. How well do you typically perform academically?

- I earn As in most or all of my classes
- I earn mostly As and Bs in my classes
- I earn mostly Bs in my classes
- I earn mostly Bs and Cs in my classes
- I earn mostly Cs in my classes
- I earn mostly Cs and Ds in my classes
- I earn mostly Ds in my classes
- I earn mostly Ds and Fs in my classes
- I am unsure of what grades I typically earn

## Footnotes

1 Though summarizing has not always been considered a generative learning strategy (see Chi & Wylie, 2014), it can be considered a generative strategy to the extent that the summary integrates prior knowledge or inference-making that goes beyond what is in the original learning material (Brod, 2021).

2 The survey included two additional questions asking students’ opinions about the effectiveness of various academic decisions (e.g., *do you learn better by completing the reading assignment before or after lecture?*). These questions were unrelated to the study strategies questions, so we do not include them in the analyses or discuss them further.

3 Pilot testing revealed that the fastest possible completion time for the survey (without reading instructions or selecting responses) was about 3 min.

4 The same significant patterns emerged whether correlations were based on Pearson or Spearman calculations.

5 It is possible that the effort involved in rereading and recopying notes (e.g., maintaining attention, physical effort if copying notes by hand) is different from the cognitive effort associated with strategies like retrieval and spacing, although we did not collect data to disambiguate this.

6 We conducted the same analysis for students who gave an effectiveness rating of 10. The pattern of results was the same, however restricting the analysis to those who gave a rating of 10 greatly limited the sample size for some of the strategies (e.g., only four students gave a rating of 10 for interleaving, and only 11 for pretesting), so we included ratings of both 9 and 10.
