# Supplementary material for: A closer look at students’ knowledge of effective learning strategies, where they learn about them, and why they do not use them
Source: Cogn Res Princ Implic. 2025 Dec 8;10:83. doi: 10.1186/s41235-025-00693-8 (PMC12686233; doi:10.1186/s41235-025-00693-8)
Supplement: Supplementary file 1 — Additional file 1. [file 41235_2025_693_MOESM1_ESM.docx]

**Supplemental Analyses**

The following tables report the analyses broken down by student major. Due to small numbers of majors in some of the non-psychology categories, data for non-psychology majors are combined such that the following tables include results for psychology majors (*n* = 84) and non-psychology majors (*n* = 101). Results across the two groups are similar and consistent with those of the full analyses.

**How Often do Students Use the Strategies?**

**Table S1A**

*How Often Psychology Majors Report Using Each Learning Strategy*

|  | Never | Rarely | Sometimes | Often | Almost Always |
| --- | --- | --- | --- | --- | --- |
| Reread | 0% | 7% | 26% | 39% | 27% |
| Highlight | 7% | 12% | 24% | 34% | 23% |
| Write Notes | 4% | 5% | 12% | 34% | 45% |
| Summaries | 8% | 30% | 25% | 29% | 8% |
| Recopy | 31% | 30% | 23% | 6% | 11% |
| Explain | 7% | 17% | 36% | 29% | 12% |
| Retrieve | 5% | 18% | 26% | 31% | 20% |
| Pretest | 21% | 30% | 24% | 16% | 10% |
| Space | 4% | 24% | 31% | 30% | 12% |
| Interleave | 6% | 12% | 42% | 32% | 8% |
| Examples | 1% | 11% | 37% | 27% | 24% |

**Table S1B**

*How Often Non-Psychology Majors Report Using Each Learning Strategy*

|  | Never | Rarely | Sometimes | Often | Almost Always |
| --- | --- | --- | --- | --- | --- |
| Reread | 2% | 5% | 29% | 31% | 34% |
| Highlight | 7% | 16% | 15% | 31% | 32% |
| Write Notes | 3% | 4% | 15% | 25% | 54% |
| Summaries | 10% | 24% | 34% | 24% | 9% |
| Recopy | 30% | 29% | 25% | 12% | 5% |
| Explain | 4% | 19% | 40% | 26% | 12% |
| Retrieve | 5% | 9% | 26% | 34% | 27% |
| Pretest | 26% | 22% | 20% | 21% | 12% |
| Space | 4% | 13% | 42% | 22% | 20% |
| Interleave | 7% | 22% | 35% | 25% | 12% |
| Examples | 4% | 12% | 41% | 31% | 13% |

**How Effective do Students Perceive the Strategies to Be?**

**Table S2A**

*Mean Effectiveness Ratings (1-10) for Each Strategy According to Psychology and Non-Psychology Majors*

|  | Reread | Highlight | Write Notes | Summaries | Recopy | Explain | Retrieve | Pretest | Space | Interleave | Examples |
| --- | --- | --- | --- | --- | --- | --- | --- | --- | --- | --- | --- |
| Psychology | 6.44 (.22) | 6.11 (.29) | 7.96 (.22) | 6.12 (.27) | 4.88 (.28) | 7.30 (.26) | 8.01 (.23) | 4.99 (.30) | 7.07 (.26) | 4.98 (.26) | 7.36 (.24) |
| Non-Psychology | 6.27 (.25) | 5.98 (.26) | 7.75 (.20) | 6.22 (.23) | 4.50 (.25) | 7.40 (.24) | 8.37 (.18) | 4.48 (.27) | 7.87 (.22) | 5.27 (.22) | 6.63 (.24) |

*Note.* Standard errors are given in parentheses.

**Do Students More Often Use Strategies that they Believe are More Effective?**

**Table S2B**

*Correlations Between Perceived Effectiveness and Frequency of Use of Each Strategy for Psychology and Non-Psychology Majors*

|  | Reread | Highlight | Write Notes | Summaries | Recopy | Explain | Retrieve | Pretest | Space | Interleave | Examples |
| --- | --- | --- | --- | --- | --- | --- | --- | --- | --- | --- | --- |
| Psychology | .19 | .68*** | .40*** | .29** | .55*** | .50*** | .38*** | .51*** | .41*** | .47*** | .57*** |
| Non-Psychology | .45*** | .66*** | .33*** | .30** | .54*** | .48*** | .51*** | .45*** | .20* | .47*** | .65*** |

*Note.* * significant at the .05 level, ** significant at the .01 level, *** significant at the .001 level.

**Why do Students Forego Using the Strategies?**

**Table S3A**

*Percentage of Psychology Majors Indicating Reasons Why They Might Not Use Each Strategy*

|  | Reread | Highlight | Notes | Summaries | Recopy | Explain | Retrieve | Pretest | Space | Interleave | Examples |
| --- | --- | --- | --- | --- | --- | --- | --- | --- | --- | --- | --- |
| Too much time | 69% | 27% | 57% | 46% | 80% | 23% | 37% | 32% | 51% | 33% | 19% |
| Does not help my learning | 13% | 38% | 13% | 19% | 25% | 18% | 8% | 29% | 12% | 37% | 24% |
| Anxious, nervous, stressed | 10% | 2% | 10% | 8% | 8% | 37% | 25% | 25% | 19% | 32% | 21% |
| Too difficult | 8% | 6% | 12% | 11% | 8% | 13% | 12% | 10% | 18% | 19% | 15% |
| Not interesting | 32% | 20% | 19% | 29% | 25% | 8% | 8% | 17% | 8% | 11% | 20% |
| Don’t know how to use it | 4% | 6% | 4% | 12% | 4% | 8% | 11% | 14% | 7% | 11% | 15% |
| Not interested in learning | 11% | 10% | 12% | 6% | 4% | 10% | 6% | 1% | 6% | 7% | 5% |
| Do not need it | 6% | 17% | 10% | 11% | 11% | 13% | 6% | 14% | 8% | 15% | 18% |
| New strategy | 0% | 1% | 4% | 13% | 6% | 11% | 6% | 12% | 10% | 8% | 11% |
| Too much effort | 29% | 17% | 34% | 30% | 43% | 17% | 25% | 20% | 29% | 12% | 20% |
| Too much planning or prep | 11% | 5% | 12% | 13% | 8% | 24% | 39% | 31% | 57% | 21% | 7% |
| Other | 6% | 11% | 11% | 7% | 2% | 15% | 7% | 7% | 4% | 7% | 13% |

*Note.* Most of the time, students used the "other" category to indicate that they do use the strategy. Occasionally they used “other" to write in circular responses that were already among the response options (e.g., it takes a lot of time; it is not very effective), and in those cases the responses were counted within those relevant response options instead of in the “other” category. "Other" responses indicating alternative reasons for not using the strategies included not having a highlighter (as a reason for not using highlighting), hands getting tired (as a reason for not taking notes), and not having anyone to explain to (as a reason for not explaining the material to someone else). Students could choose more than one response for each strategy, so percentages do not sum to 100.

**Table S3B**

*Percentage of Non-Psychology Majors Indicating Reasons Why They Might Not Use Each Strategy*

|  | Reread | Highlight | Notes | Summaries | Recopy | Explain | Retrieve | Pretest | Space | Interleave | Examples |
| --- | --- | --- | --- | --- | --- | --- | --- | --- | --- | --- | --- |
| Too much time | 71% | 28% | 53% | 53% | 76% | 26% | 36% | 30% | 50% | 22% | 15% |
| Does not help my learning | 22% | 34% | 5% | 21% | 35% | 13% | 3% | 36% | 5% | 19% | 27% |
| Anxious, nervous, stressed | 13% | 6% | 10% | 5% | 6% | 36% | 28% | 30% | 19% | 26% | 7% |
| Too difficult | 9% | 3% | 8% | 14% | 9% | 8% | 14% | 12% | 13% | 23% | 18% |
| Not interesting | 31% | 17% | 11% | 17% | 21% | 13% | 10% | 11% | 9% | 6% | 16% |
| Don’t know how to use it | 3% | 6% | 1% | 10% | 5% | 1% | 5% | 9% | 4% | 10% | 18% |
| Not interested in learning | 6% | 5% | 6% | 4% | 4% | 2% | 7% | 6% | 4% | 3% | 4% |
| Do not need it | 5% | 16% | 6% | 9% | 15% | 9% | 4% | 9% | 4% | 11% | 10% |
| New strategy | 3% | 3% | 2% | 7% | 6% | 4% | 5% | 12% | 3% | 15% | 9% |
| Too much effort | 35% | 24% | 35% | 43% | 48% | 24% | 32% | 19% | 27% | 21% | 15% |
| Too much planning or prep | 8% | 3% | 6% | 11% | 8% | 14% | 31% | 25% | 57% | 18% | 6% |
| Other | 3% | 9% | 9% | 3% | 2% | 18% | 5% | 4% | 5% | 4% | 7% |

*Note.* Most of the time, students used the "other" category to indicate that they do use the strategy. Occasionally they used “other" to write in circular responses that were already among the response options (e.g., it takes a lot of time; it is not very effective), and in those cases the responses were counted within those relevant response options instead of in the “other” category. "Other" responses indicating alternative reasons for not using the strategies included not having a highlighter (as a reason for not using highlighting), hands getting tired (as a reason for not taking notes), and not having anyone to explain to (as a reason for not explaining the material to someone else). Students could choose more than one response for each strategy, so percentages do not sum to 100.

**Where do Students Learn About the Strategies?**

**Table S4A**

*Percentages of Psychology Majors Indicating Where They Learned about Each Strategy*

|  | Reread | Highlight | Notes | Summaries | Recopy | Explain | Retrieve | Pretest | Space | Interleave | Examples |
| --- | --- | --- | --- | --- | --- | --- | --- | --- | --- | --- | --- |
| Never learned | 1% | 2% | 1% | 13% | 15% | 11% | 0% | 17% | 2% | 31% | 8% |
| Teacher | 57% | 52% | 70% | 60% | 34% | 39% | 75% | 60% | 66% | 33% | 49% |
| Tutor | 6% | 17% | 11% | 7% | 10% | 11% | 14% | 8% | 8% | 4% | 5% |
| Friends | 17% | 29% | 23% | 11% | 25% | 27% | 30% | 15% | 8% | 11% | 10% |
| Social media | 4% | 8% | 6% | 8% | 12% | 8% | 11% | 7% | 7% | 5% | 7% |
| Research | 6% | 6% | 7% | 4% | 5% | 11% | 12% | 6% | 21% | 5% | 11% |
| Academic center | 4% | 5% | 7% | 5% | 4% | 5% | 12% | 4% | 13% | 4% | 4% |
| Own experience | 49% | 39% | 52% | 21% | 23% | 42% | 33% | 20% | 32% | 26% | 40% |
| Do not remember | 11% | 13% | 8% | 6% | 14% | 12% | 7% | 8% | 10% | 12% | 12% |
| Other | 1% | 1% | 2% | 2% | 2% | 0% | 1% | 0% | 1% | 0% | 0% |

*Note.* Students listed parents as a common “other” response, and sometimes also listed television. Occasionally students used “other" to write in circular responses that were already among the response options (e.g., I just tried it on my own), and in those cases the responses were counted within those relevant response options instead of in the “other” category. Students could choose more than one response for each strategy, so percentages do not sum to 100.

**Table S4B**

*Percentages of Non-Psychology Majors Indicating Where They Learned about Each Strategy*

|  | Reread | Highlight | Notes | Summaries | Recopy | Explain | Retrieve | Pretest | Space | Interleave | Examples |
| --- | --- | --- | --- | --- | --- | --- | --- | --- | --- | --- | --- |
| Never learned | 0% | 1% | 0% | 8% | 19% | 5% | 1% | 16% | 4% | 25% | 15% |
| Teacher | 61% | 65% | 69% | 50% | 29% | 50% | 69% | 60% | 69% | 39% | 44% |
| Tutor | 13% | 17% | 15% | 11% | 6% | 12% | 15% | 11% | 15% | 4% | 8% |
| Friends | 25% | 30% | 32% | 14% | 14% | 37% | 34% | 11% | 24% | 8% | 14% |
| Social media | 10% | 10% | 17% | 6% | 6% | 15% | 16% | 4% | 15% | 9% | 8% |
| Research | 3% | 3% | 8% | 2% | 3% | 7% | 8% | 7% | 12% | 10% | 8% |
| Academic center | 5% | 6% | 9% | 7% | 7% | 4% | 10% | 2% | 16% | 5% | 6% |
| Own experience | 46% | 34% | 40% | 25% | 32% | 42% | 34% | 18% | 32% | 16% | 33% |
| Do not remember | 7% | 9% | 9% | 13% | 16% | 12% | 8% | 7% | 7% | 15% | 10% |
| Other | 0% | 1% | 0% | 1% | 0% | 1% | 0% | 2% | 1% | 0% | 1% |

*Note.* Students listed parents as a common “other” response, and sometimes also listed television. Occasionally students used “other" to write in circular responses that were already among the response options (e.g., I just tried it on my own), and in those cases the responses were counted within those relevant response options instead of in the “other” category. Students could choose more than one response for each strategy, so percentages do not sum to 100.

**Is Academic Performance Related to Strategy Use?**

**Table S5**

*Correlations (Spearman’s ρ) Between Perceived Effectiveness and Academic Performance for Each Strategy for Psychology and Non-*

*Psychology Majors*

|  | Reread | Highlight | Write Notes | Summaries | Recopy | Explain | Retrieve | Pretest | Space | Interleave | Examples |
| --- | --- | --- | --- | --- | --- | --- | --- | --- | --- | --- | --- |
| Psychology | -.13 | -.21 | .10 | -.14 | -.16 | .18 | .21 | .00 | .05 | .08 | .16 |
| Non-Psychology | -.14 | .04 | -.06 | .01 | -.08 | .15 | .09 | -.07 | .00 | -.10 | .09 |

*Note.* No observed correlations were statistically significant (all *p*s >.05). The non-parametric Spearman’s *ρ* was used due to the non-normal distribution of reported academic performance (86% of students reported earning As or Bs in most of their classes). As these students are likely to be early in their college career, this could be a result of students reporting academic performance from high school or other academic settings, potentially leading to the inflation of academic performance reported here.
